# Sleep Loss as a Factor to Induce Cellular and Molecular Inflammatory Variations

**DOI:** 10.1155/2013/801341

**Published:** 2013-12-03

**Authors:** Gabriela Hurtado-Alvarado, Lenin Pavón, Stephanie Ariadne Castillo-García, María Eugenia Hernández, Emilio Domínguez-Salazar, Javier Velázquez-Moctezuma, Beatriz Gómez-González

**Affiliations:** ^1^Area of Neurosciences, Department of Biology of Reproduction, CBS, Universidad Autónoma Metropolitana, Unidad Iztapalapa, Avenida San Rafael Atlixco No. 186, Colonia Vicentina, Iztapalapa, 09340 Mexico City, Mexico; ^2^Department of Psychoimmunology, National Institute of Psychiatry, “Ramón de la Fuente”, Calzada México-Xochimilco 101, Colonia San Lorenzo Huipulco, Tlalpan, 14370 Mexico City, DF, Mexico

## Abstract

A reduction in the amount of time spent sleeping occurs chronically in modern society. Clinical and experimental studies in humans and animal models have shown that immune function is impaired when sleep loss is experienced. Sleep loss exerts a strong regulatory influence on peripheral levels of inflammatory mediators of the immune response. An increasing number of research projects support the existence of reciprocal regulation between sleep and low-intensity inflammatory response. Recent studies show that sleep deficient humans and rodents exhibit a proinflammatory component; therefore, sleep loss is considered as a risk factor for developing cardiovascular, metabolic, and neurodegenerative diseases (e.g., diabetes, Alzheimer's disease, and multiple sclerosis). Circulating levels of proinflammatory mediators depend on the intensity and duration of the method employed to induce sleep loss. Recognizing the fact that the concentration of proinflammatory mediators is different between acute and chronic sleep-loss may expand the understanding of the relationship between sleep and the immune response. The aim of this review is to integrate data from recent published reports (2002–2013) on the effects of sleep loss on the immune response. This review may allow readers to have an integrated view of the mechanisms involved in central and peripheral deficits induced by sleep loss.

## 1. Introduction

Sleep is a vital phenomenon, classically divided into two distinct phases: sleep with rapid eye movements (REM) and sleep without rapid eye movements (non-REM) [1]. In humans, three stages of non-REM sleep have been characterized by electroencephalography (EEG); these include low-frequency slow wave sleep (SWS) with EEG synchronization, light sleep, and an intermediate sleep stage 2. In contrast, REM sleep is characterized by EEG activity similar to that of waking and by the loss of muscle tone [2, 3]. Both phases, REM sleep and non-REM sleep, alternate throughout total sleep time [2, 3]. REM sleep is amply studied because it is considered important for learning, memory consolidation, neurogenesis, and regulation of the blood-brain barrier function [4–8], while non-REM sleep is related to hormonal release (e.g., growth hormone secretion), the decline in the thermal set point, and is characterized by a reduction of cardiovascular parameters (e.g., lowering of blood pressure) [9, 10]. Although sleep constitutes a considerable portion of the mammalian lifetime [2], specific sleep function still remains controversial. Many hypotheses have been proposed, including tissue repair, thermoregulation, homeostatic restoration, memory consolidation processes, and preservation of neuro-immune-endocrine integrity [10, 11].

The paramount role of sleep in the physiology of animal models and humans is evident by the effects of sleep loss. Serious physiological consequences of sleep loss include emotional reactivity, cognitive dysfunction (deficits in learning, memory, and decision making), decreased neurogenesis, and metabolic disturbances that may result in the death of experimental animals [1, 7, 12–14]. Sleep loss effects can be evaluated by several methodologies, including acute total or selective sleep deprivation and sleep restriction (also called partial sleep deprivation) or sleep fragmentation. In some cases, deprivation devices connected to the electroencephalograph have been used to selectively deprive a specific sleep phase. In humans, total sleep deprivation is common in individuals working more than 24 hours continuously, while sleep restriction is defined as diminution of time spent asleep. Sleep restriction is linked to lifestyle including longer work hours and shift-work and increased accessibility to media of all sorts, or medical conditions such as insomnia [15, 16]. Pathological conditions (e.g., obstructive sleep apnea (OSA), drug addiction) and aging have a common pattern of sleep fragmentation (also called sleep disruption) characterized by numerous awakenings despite normal time spent asleep [16]. Most of the current knowledge on the effects of sleep loss in humans comes from studies of total sleep deprivation applied for brief time periods or partial sleep deprivation (2-3 hours less than normal sleep time) for one night or even for chronic periods [15, 16]. The majority of animal models used to study the physiological effects of sleep loss are based primarily on total sleep deprivation [16]. Although this method does not resemble human conditions, it still provides valuable information on sleep loss effects.

To study the relationship between sleep and the immune system, researchers have relied on two basic approaches; in the first approach, human volunteers or animals (mainly rodents) are subjected to the administration of immune-stimulating substances (or pathogen administration in animals), and the effects of these manipulations on sleep are evaluated. In the second approach, human volunteers or animals are subjected to sleep loss protocols (sleep deprivation, sleep restriction, or sleep fragmentation) and immunological products such as cells and/or soluble mediators are measured. Here, we present a compilation of recent evidence about the effects of sleep loss on the immune system in both humans and rodents, under acute and chronic sleep loss. Additionally, we propose how sleep recovery might restore the normal balance between proinflammatory and anti-inflammatory molecules at the systemic level and how immune mediators might be in direct contact with the central nervous system via blood-brain barrier disruption, modifying neural activity and the possible pathway for neurological impairments.

## 2. Sleep Loss as a Stressful Factor

Sleep loss has been deemed a stressor [17, 18]; however, sleep and stress differ in the profile of circulating molecules and in their effects on the immune system. Stress is the response of the organism to any stimulus that alters the homeostasis [19]. The adverse stimuli generating stress, either physical or psychological, also vary in their temporal dimension. Acute stress occurs when stressors appear once and remain for a short period of time (some minutes or hours); while, chronic stress occurs when stressors are repetitive and long lasting (appearing for weeks or months) [19]. Since the initial description of the phenomenon [20], it has been shown that stressors induce activation of the hypothalamus-pituitary-adrenal (HPA) axis and of the sympathetic nervous system [19, 21]. At the beginning of the stress response, there is a large sympathetic activation, followed by glucocorticoid release from the adrenal cortex. Over a prolonged stress period, the adrenaline response is rapidly habituated; however, glucocorticoids remain elevated only when stressors are unpredictable and uncontrollable. If the subject is capable of predicting the appearance of chronic stressors and has control over them, the glucocorticoid response also disappears [21].

Regarding the effect of stress on the immune system, it has been shown that acute stress has an immunostimulatory effect; adrenaline increases the circulating numbers of neutrophils, macrophages, natural killers, and lymphocytes, while glucocorticoids promote traffic of leukocytes to the skin, mucosal lining of the gastrointestinal and urinary-genital tracts, the lung, and liver, both in humans and in experimental animal models [22–27]. Therefore, acute stress seems to prepare the immune system to cope with the damage induced by the noxious agent. On the contrary, chronic stress suppresses the immune function by modifying the levels of both proinflammatory (e.g., interleukin (IL)-6 and tumor necrosis factor (TNF)-*α*) and anti-inflammatory cytokines (e.g., IL-10, IL-4) [28], by reducing the numbers and traffic of leukocytes [27], and by up-/downregulating T cell number and function [29]. Specifically, glucocorticoids act on antigen-presenting cells (APCs) and T helper 1 (T_h_1) cells, inhibiting their production of IL-12, interferon (IFN)-*α*, IFN-*β*, and TNF-*α*, but upregulating the production of anti-inflammatory cytokines (IL-4, IL-10, and IL-13) by T_h_2 cells [30].

Since the pioneer studies, sleep loss has been tightly linked to stress; in the first studies it was shown that sleep deprived animals had larger adrenals than their counterpart controls [1, 31]. In animal models, the classical methods for sleep deprivation consist of highly aversive environments (e.g., water surrounding small platforms); therefore, additional animals subjected to the aversive environment but without any sleep loss are constantly included as controls for the procedure. The measurement of circulating levels of glucocorticoids is the main stress index; nevertheless, depending on the intensity and duration of sleep loss, cortisol/corticosterone levels may increase [32–35], not change [33, 36], or even decrease [37] (see Table 1). It is known that the chronic increase in cortisol/corticosterone levels desensitize glucocorticoid receptors, promoting an altered control of the HPA axis [38]; this may explain the maintenance or even the decrease in glucocorticoid levels after sleep deprivation (e.g., >40 h in humans) [39] or chronic sleep restriction (e.g., 21 days in rats) [33].

The role of glucocorticoids in sleep homeostasis has been carefully studied; glucocorticoid administration in both humans and animal models induces waking EEG activity (e.g., [42, 43]); in addition, glucocorticoid administration decreases REM sleep and promotes SWS in humans [42] and decreases SWS and increases sleep latency in animal models [42]. When they occur, increased corticosterone levels secondary to sleep deprivation are unnecessary for sleep recovery; in animal models, a large sleep rebound was observed after acute sleep deprivation, despite adrenalectomy [44]. Moreover, under chronic REM sleep deprivation in rats, where corticosterone levels are similar to basal levels [33], a tendency to REM sleep rebound is also observed [45].

In the vast majority of studied phenomena (e.g., studies of sleep loss effects on hippocampal neurogenesis), it has also been found that sleep loss effects are maintained even in animals subjected to adrenalectomy [46]. Additionally, chronic administration of an inhibitor of corticosterone secretion (metyrapone) in REM sleep deprived animals did not revert memory deficits; hence glucocorticoids are not responsible for the memory impairments associated to REM sleep loss [47]. These data show that sleep loss may cause more functional deficits than those caused by stress only. It is very likely that the effects of REM sleep deprivation on neural, endocrine, and immune systems accumulate throughout the experimental procedure without any opportunity to restore homeostasis by adequate sleep recovery. Notwithstanding, some authors still consider that sleep loss is a stressful event [18], while the vast majority of sleep researchers deem sleep deprivation and stress as independent events [42–44, 46–48].

## 3. Sleep and the Immune Response

It is well known that sleep loss makes an individual more susceptible to disease and, conversely, that sleep is important for recovery from illness. Specific immunological active peptides or neuroendocrine hormones influence the sleeping-waking brain, and sleep disturbances may affect inflammatory components. Cellular (macrophages, neutrophils, eosinophils, basophils, natural killer, and T and B lymphocytes) and molecular (proinflammatory cytokines and acute phase proteins) inflammatory components that act as mediators of the acute phase response in inflammatory diseases, additionally, play a role as modulators of metabolic functions that involve the central nervous system, including sleep.

### 3.1. Effects of Inflammatory Components on Sleep

Cytokines that affect sleep in both humans and laboratory animals include IL-1*α*, IL-1*β*, IL-2, IL-4, IL-6, IL-8, IL-10, IL-13, IL-15, IL-18, TNF-*α*, TNF-*β*, IFN-*α*, IFN-*β*, IFN-*γ*, and macrophage inhibitory protein (MIP)-1*β* (MIP-1*β*) [49]. Immune signaling molecules such as cytokines are present in the healthy brain, where they interact with neurochemical systems (e.g., serotoninergic, cholinergic, and glutamatergic systems) [49, 50] to regulate normal sleep. Particularly, IL-1*α*, IL-1*β*, and TNF-*α* have been widely investigated to state that they are involved in the regulation of physiological sleep. Signaling receptors for both IL-1 (*α* and *β*) and TNF-*α* are present in brain areas involved in sleep physiology including the hypothalamus, brainstem, hippocampus, and cerebral cortex [49]. The brain interacts with peripheral inflammatory mediators through the innervation of lymphoid tissues or the transport or action of these molecules on the blood-brain barrier [51]. In addition, glial cells such as microglia and astroglia, as well as pericytes are capable of releasing proinflammatory mediators in response to peripheral signals (chemokines, acute phase proteins, nitric oxide, and adenosine) contributing to the action of inflammatory mediators upon neuronal function [52, 53]. Because IL-1*α*, IL-1*β*, and TNF-*α* are the most studied cytokines involved in sleep regulation, we focus mainly on these three cytokines; however, the role of IL-6 will also be reviewed because this proinflammatory cytokine is highly related to the interaction between sleep loss and the immune response.

#### 3.1.1. Effect of Proinflammatory Cytokine on Sleep in Humans

Interleukin-1 is a key mediator of the acute phase response in an infected host [54]. IL-1*α* and IL-1*β* together with TNF-*α* have many physiological roles, such as in cognition, synaptic plasticity, and immune function. Both IL-1*β* and TNF-*α* are also well-characterized as to their actions on sleep regulation [55]. For instance, IL-1*β* is a potent enhancer of non-REM sleep that induces symptoms associated with sleep loss such as sleepiness, fatigue, and poor cognition [56].

Under pathological conditions (e.g., cancer, multiple sclerosis) cytokine administration is used as a treatment [57, 58] and sleep patterns are altered [59, 60]; in patients with multiple sclerosis numerous sleep pathologies (e.g., insomnia, hypersomnia, circadian rhythm sleep disorders, and movement- and breathing-related sleep disorders) have been described [59], while in cancer patients complaints about sleep fragmentation and insomnia are frequent [60]. Although sleep disturbances are frequently reported in autoimmune pathologies and mood disorders with an inflammatory component [59–61], the aetiology of sleep alterations remains unclear. To cite a few instances, it has been reported that autoimmune diseases that exhibit autoantibodies against neuronal voltage-gated potassium channel (VGKC) complexes such as limbic encephalitis or Morvan syndrome present sleep disturbances like insomnia, REM sleep behavior disorder, hypersomnia, and somniloquy [62, 63]. Interestingly, immunotherapy in patients with autoimmune diseases promotes significant sleep improvement in 80% of patients [62]. Also, infections with a proinflammatory component induce sleep disorders, up to 70% of persons living with human immunodeficiency virus (HIV) experience sleep disturbances including insomnia and obstructive sleep apnoea (OSA) syndrome [64], and in people affected by leprosy the prevalence of restless leg syndrome is higher than the general population [65]. In the same way, inhibition of proinflammatory cytokine signalling has been proposed as a viable strategy for targeting sleep disturbances in patients with evidence of proinflammatory activity [66]. For instance, in alcohol-dependent males, inflammatory markers correlated with REM sleep increase [66], but the pharmacological neutralization of TNF-*α* by etanercept (a decoy receptor that binds to TNF-*α*) reduced REM sleep until normal values [67]. In addition, both IL-1 (*α* and *β*) and TNF-*α* are present in a variety of clinical conditions involving sleep disorders, such as chronic insomnia and OSA (reviewed in [68]).

#### 3.1.2. Effect of Proinflammatory Cytokines on Sleep in Animal Models

It has been known for over 50 years that mammalian cerebrospinal fluid contains sleep-promoting substances that accumulate during wakefulness [10]. The common criteria to consider any substance a somnogenic molecule include (1) whether the substance injected enhances sleep, (2) whether sleep is reduced if the substance is inhibited, and (3) whether the substance is altered in pathological states associated with sleep disorders. All of these criteria have been met by IL-1*α*, IL-1*β*, IL-6, and TNF-*α* [68].

The effects of IL-1*α*, IL-1*β*, and TNF-*α* on sleep was reported in several animal species including rodents, monkeys, cats, and sheep. Induction of non-REM sleep by IL-1*α*, IL-1*β*, and TNF-*α* is independent of the route of administration (e.g., intracerebroventricular (ICV), intraperitoneal, subcutaneous) and its effect is dose-dependent [68, 69]. In rodents, classical studies show that low doses of IL-1*β*, through ICV administration, increase non-REM sleep when it is administered during the light phase [69]. However, IL-1*α* or IL-1*β* also induce non-REM sleep fragmentation [49], and high doses of IL-1*β*, administered during the dark phase, suppress non-REM sleep [68].

In addition to the pioneer studies on sleep regulation by cytokines, recent studies focus on the molecular pathways involved in physiological sleep regulation. Recently, mice lacking the TNF 55kDa receptor (TNFR-KO) present a decrease in the amount of non-REM and REM sleep [70]. Furthermore, experimental studies in rodents show that proinflammatory cytokine-induced sleep disturbances can be reversed by administration of anti-inflammatory cytokines or specific cytokine antagonists (e.g., IL-1 receptor antagonist, IL-1ra) [68]. The strong relationship between sleep and its modulation by proinflammatory cytokines provides a key to understand how sleep loss is capable of altering the immune system and subsequently promotes metabolic, cardiovascular, and neurodegenerative impairments [15].

### 3.2. Effect of Sleep Loss on Immunological Response in Humans

#### 3.2.1. Effects of Sleep Loss on Cellular Immune Components

Circadian rhythms have been described for white blood cells (WBC) in humans; numbers of circulating natural killers (NK) and neutrophils peak at midday and show a nadir during the night; while, monocytes, T and B lymphocytes peak during the first half of the night and present the lowest values during the day hours [71]. Sleep loss shifts the normal circadian rhythm of WBC. In 24-hour total sleep deprived humans, monocytes, T and B lymphocytes presented a delay in the zenith of the rhythm with attainment of peak values between 3 and 6 hours later than in normal sleep conditions [71]; while the rhythm of NK flattened with a net increase in the NK number during the sleep deprived night as compared to normal sleep conditions [71, 72]. However, only few human studies have repeatedly drawn blood samples from sleep deprived subjects to measure circadian effects of sleep deprivation on WBC counts; the majority of reported studies quantify circulating WBC only once, on the morning after sleep deprivation and compare those values with normally sleeping subjects. Generally, in those studies leukocyte population increases after acute sleep deprivation, mainly by rises in circulating numbers of monocytes and neutrophils; in contrast, circulating numbers of B and T lymphocytes remain stable immediately after sleep loss, but exhibit changes after sleep recovery (see Table 2) [73, 74]. Sleep restriction to 4 hours in bed during 5 consecutive nights decreased the number of circulating NK and increased the number of B lymphocytes, maintaining stable the numbers of other WBC [75]. Differences among these studies may be explained by the different techniques to draw blood samples, such as sex, race, or age of the participants.

#### 3.2.2. Effects of Sleep Loss on Molecular Inflammatory Component

*Effect of Sleep Loss on Antibodies.* Few studies have examined the consequences of sleep loss on the immune response to vaccination in healthy individuals; highly variable findings have been reported [78–80]. Total sleep deprivation during one night prior to hepatitis A vaccination reduced specific antibody titters in the long-term (28 days postvaccination) in both males and females [78]. However, the same 24 hours of total sleep deprivation reduced specific antibody titters to influenza AH1N1 virus vaccine only in males in the short term (5 days post-vaccination), while sleep deprived females did not have a significant difference as compared to normal sleeping subjects [79]. In another study, short sleep durations during the week of hepatitis B vaccination decreased viral specific antibody titters in both male and female volunteers; while the contrary was true, higher levels of antibody titters were observed in participants with long sleep durations during the week of vaccination [80]. Although few, those studies suggest that sleep plays an important role in humoral immunity, especially in antibody production; however, more studies are necessary to elucidate how sleep loss may induce changes in cellular immune components and subsequently induce antigen-specific immune impairments, such as insufficient antibody production.

*IL-1*α*, IL-1*β*, IL-6, and TNF-*α*: The Most Studied Cytokines under Sleep Loss Conditions.* Human studies that evaluate sleep loss effects have focused on the correlation among inflammatory markers and metabolic and cardiovascular diseases. For instance, in a study with 124 healthy volunteers, inflammatory markers, such as endothelin-1 (ET-1) and IL-6, were associated with an increase in total sleep time and REM sleep latency [81]. These results show that poor sleep is directly associated with inflammatory status. In the same way, shorter sleep duration is also related to obesity and cardiovascular diseases [82]. It is known that obesity, diabetes, and cardiovascular diseases share a common mechanism characterized by the inflammatory process. If sleep loss induces low-intensity inflammation, we may consider that sleep loss is associated with metabolic and cardiovascular disease generation through immunological deregulation [15].

Similar to immune cells, cytokine production presents circadian rhythms; proinflammatory cytokines present a peak in early nocturnal sleep in correlation with the accumulation of molecules such as adenosine or reactive oxygen species that promote proinflammatory cytokine release; however the dominance of the proinflammatory response shifts during late sleep, when REM sleep is present, promoting the production of anti-inflammatory cytokines [51, 83]. The different periods of exposure to proinflammatory mediators might explain the reported differences between cytokine plasma levels in sleep loss protocols.

Sleep deprivation protocols, lasting 40–88 hours in humans, induce controversial changes in plasma levels of IL-1*α*, IL-1*β*, IL-6, and TNF-*α*, with reported findings of increases, decreases, or absence of measurable changes in cytokine levels [34, 39–41, 83, 84] (see Table 3). For example, IL-6 plasma levels increased after one week of sleep restriction in healthy males [41]. In contrast, a study with 40 hours of continuous total sleep deprivation found decreased IL-6 levels in healthy men [40]. These discrepancies may be attributed to the method employed to obtain blood samples; intravenous catheters used for repetitive blood sampling increase local IL-6 production, which might confound the sleep-dependent changes in plasma concentrations of this cytokine [85]. In addition, all the cellular sources of proinflammatory cytokines are not known, although monocytes, which make up about 5% of circulating leukocytes, are major contributors to proinflammatory cytokine production in peripheral blood [71]. Interestingly, studies reported differences in proinflammatory cytokine levels independent of WBC number or activity. This may be explained by considering other sources of cytokines (e.g., macrophages in adipose tissue, epithelium, and endothelium) [86], which may also be affected by sleep loss.

In addition to modifying IL-1*α*, IL-1*β*, IL-6, TNF-*α*, and IL-17A levels, five nights of sleep restriction are accompanied by increased heart rate; both proinflammatory cytokines and hypertension are important risk factors for development of cardiovascular disease [75, 97]. IL-17A plays a key role in sustaining tissue damage in the brain, heart, and intestine, sometimes promoting the development of autoimmune diseases [75]. Helper T cells producing IL-17A require activation by IL-6 [98]. Interestingly, IL-17A is a potent inducer of C-reactive protein (CRP) expression in hepatocytes and in coronary artery smooth muscle cells [99] (see next section). The combination of circulating cytokines with other inflammatory mediators achieves a low-grade inflammatory status induced by sleep loss.

*Effect of Sleep Loss on Acute Phase Proteins. *The effects of sleep loss on acute phase proteins are poorly studied. For instance, acute total sleep deprivation (one night) results in elevated high-sensitivity C-reactive protein (hsCRP) concentrations, which is a stable marker of inflammation that has been shown to be predictive of cardiovascular morbidity [89]. CRP production in the liver is stimulated by proinflammatory cytokines such as IL-6 or IL-17, which are highly expressed after sleep loss periods [75]. CRP is an important inflammatory marker because this protein lacks diurnal variations [15, 100]. In contrast, total sleep deprivation for 40 hours in young adults decreased CRP levels while increasing other inflammatory markers such as E-selectin and the intracellular adhesion molecule (ICAM)-1 [81]. Several methodological differences among the studies may contribute to the inconsistent findings for CRP (see Table 3), including the sleep deprivation period, blood sampling frequency, nutrition, and all effects and differences in subject's characteristics such as body mass index (BMI), because obesity increases proinflammatory markers [101]. In addition to voluntary sleep loss, several health conditions (e.g., pregnancy, depression) may contribute to deregulation of the immune system [102].

#### 3.2.3. Sleep Loss and Depression

Recently, it has been suggested that one of the functions of sleep may be to regulate the neuro-immune-endocrine network [11]. In this regard, an excellent example of the interaction between the neuro-endocrine-immune network and sleep disorders is major depressive disorder, which is characterized by high levels of cortisol and TNF-*α*, increased NK percentages, diminished B lymphocyte counts, and no significant variations in T lymphocytes [103]; these changes are similar to the effects observed after sleep deprivation (see previous sections). In depressed patients, sleep disturbances include intermittent awakenings, prolonged sleep latency, and shortened REM sleep latency, which represent sleep fragmentation or sleep restriction (in the case of insomnia) [104, 105]. All antidepressants affect sleep architecture and quality [104], and the immune system might be altered in long-term treatment periods. For instance, depressed patients treated with selective serotonin-reuptake inhibitors for 20 weeks showed an increase in B lymphocytes [106]. The role of both major depressive disorder and sleep disturbances on the increased risk to develop metabolic disturbances is discussed in another recent review (please see [107]).

### 3.3. Effect of Sleep Loss on the Immune System in Animal Models

#### 3.3.1. Effects of Sleep Loss on Cellular Immune Components

As in humans, the circadian oscillation of immune cells and molecules in rodents has been described. In mice, Ly6C^hi^ inflammatory monocyte traffic is regulated by the circadian gene Bmal1, and is higher during the resting phase and decreases during the active phase [108, 109]. Macrophages and NK contain a cell-autonomous circadian clock [110, 111]. In addition, T lymphocytes exhibit clock gene regulation, mice immunized during the light phase show a stronger specific T lymphocyte response than those immunized during the dark phase [112]. These data suggest that a disruption of circadian rhythms might be related with changes in the WBC count after sleep loss. In rodents subjected to selective REM sleep deprivation for 24 and 240 hours, the number of T lymphocytes decreases and of B lymphocytes does not change. In the same experiment, an increase in NK percentage was observed [25]. Similarly, REM sleep deprivation for 96 hours does not promote changes in number of lymphocytes but it does increase the number of monocytes and neutrophils [33]. Controversially, REM sleep restriction promotes a decrease of leukocyte number [33]. These contradictory findings might be explained by the alteration in clock genes involved in the circadian oscillation of WBC.

#### 3.3.2. Effects of Sleep Loss on the Molecular Inflammatory Component

Similar to humans, rodents subjected to sleep loss exhibit a proinflammatory component characterized by increase in proinflammatory cytokines, namely IL-1, IL-6, IL-17, and TNF-*α* as compared to control animals [32, 33]. The proinflammatory status after sleep loss may be explained, in part, because the alteration in clock genes of monocytes is associated with the upregulation of proinflammatory cytokines via NF-*κ*B activation [76, 113]. Exposure to proinflammatory cytokines in chronic sleep restriction may promote tissue damage and subsequent loss of function; however, acute sleep deprivation may exert beneficial effects on the immune system. For instance, acute sleep deprivation is associated with a reduction in ischemia-induced IL-1*β* gene expression and attenuation of neuronal damage in the hippocampus. This finding may be explained by increased gene expression of IL-6 and the anti-inflammatory cytokine IL-10 after sleep deprivation [114].

## 4. Impact of Sleep Recovery on Sleep Loss-Induced Inflammation

Usually, the modification of cellular immune components and molecular inflammatory markers by sleep loss returns to basal levels after sleep recovery periods [34, 76]. However, depending on sleep loss time, some immune components may remain altered after sleep recovery or may even present alterations only after sleep recovery [32, 33, 100, 115]. For instance, monocyte and neutrophil numbers do not change after REM sleep deprivation in rats for 96 hours; however, after 24 hours of sleep recovery, monocyte and neutrophil numbers increase in comparison to control animals [33]. Levels of other WBC in rats decrease immediately after sleep restriction, but 24 hours of uninterrupted sleep restores the basal levels [33]. Like cellular components, molecular inflammatory mediators are altered after sleep recovery. Plasma levels of complement protein C3 were higher than controls after sleep deprivation in rats and remained elevated after sleep recovery [33]. REM sleep deprivation in rats (72 hours) increases plasma levels of IL-1, IL-6, IL-17A, and TNF-*α*. Proinflammatory cytokines IL-1*α*, IL-1*β*, and IL-6 return to basal levels after sleep recovery, whereas IL-17A and TNF-*α* remain higher than controls even after one week of normal sleep [32]. In addition, in the same study anti-inflammatory cytokines, such as IL-10, do not increase. Within the same context, in humans, increased sleepiness after sleep restriction was better reversed with a nap or with extended sleep recovery conditions (10 hours of uninterrupted sleep) [36]. In addition, other parameters associated with sleep loss were restored; for example, cortisol decreased immediately after a nap [36]. A midday nap prior to recovery sleep or an extended night of sleep can return leukocyte counts to baseline values [36]. Although long periods of sleep appear to be the solution to restore immune function, it has been reported that sleeping more than 9 hours is related with greater physical decline than midrange or short periods of sleep and also is related with increased risk of mortality associated with cardiovascular impairments [116].

## 5. Sleep Loss Alters the Blood-Brain Barrier

Up to this time, we have only discussed the effect of sleep loss on immune mediators at the peripheral level. Nevertheless, brain-immune system communication is very complex and it includes the direct action of proinflammatory cytokines synthesized in the brain [52, 117, 118] on neuronal systems, or the effect of peripheral cytokines on blood-brain barrier components [51]. We reported that chronic REM sleep restriction in rats induces blood-brain barrier disruption and that brief sleep recovery periods lessened these effects in several brain regions. Nevertheless, in the hippocampus hyperpermeability remained even after sleep opportunity [8]. These findings suggest that if sleep restriction increases the unselective transportation across the blood-brain barrier, proinflammatory mediators and toxic blood-borne molecules might enter the brain promoting neurochemical changes or excitotoxicity events that may explain cognitive and emotional impairments associated with sleep deficits.

## 6. Conclusion and Future Directions

**Figure 1:**
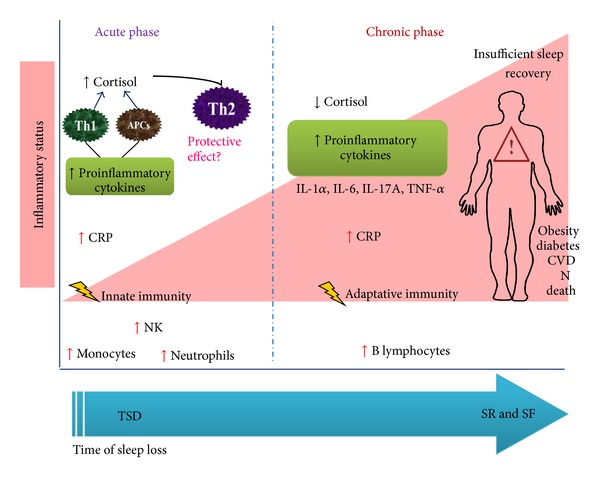
Sleep loss promotes a low-grade proinflammatory status. Sleep loss is characterized by an increase in circulating proinflammatory cytokines (IL-1*β*, IL-6, IL-17A, TNF-*α*) and CRP. Image shows the differential effect of sleep loss on the immune system after acute total sleep deprivation and prolonged sleep restriction and or sleep fragmentation. The acute and chronic events of sleep loss correlate with the temporal immune response (innate and adaptive). Prolonged sleep loss plus insufficient sleep recovery are considered an important risk factor to develop metabolic, cardiovascular, and neurodegenerative diseases related with the deregulation of the neuro-endocrine-immune network. Abbreviations: APCs; antigen-presenting cells; CRP, C-reactive protein; CVD, cardiovascular disease; N, neurodegenerative diseases; NK, natural killer; SR, sleep restriction; SF, sleep fragmentation; TSD, total sleep deprivation.

Recent studies focus on evaluating the correlation between inflammatory markers and sleep disorders. Conditions such as obesity or infections may exacerbate the inflammatory condition contributing to systemic impairments and susceptibility to pathogens. Although sleep recovery may restore immune system alterations, when sleep loss is prolonged the proinflammatory status may remain and promote neuro-immune-endocrine axis disruption. Constant systemic inflammatory status after prolonged wakefulness may be the source of metabolic, cardiovascular, and cognitive impairments. The immune system is altered by sleep loss; however, more studies are necessary to elucidate how sleep loss promotes the release of inflammatory mediators and how these molecules act on the brain promoting local and systemic alterations that exacerbate the proinflammatory status and contribute to sleep disorders, fostering a vicious circle between inflammation and sleep disturbances (see Figure 1).

In the last few years, several reviews on sleep and immunity have been written. A review of some of their conclusions could be relevant. Some of them conclude that sleep modulates and is modulated by inflammation [15, 119], or that sleep deprivation impairs immune function, particularly the immune memory/humoral immune response [15, 51]. Also, some of them work with the hypothesis that sleep deprivation is a type of stress and that glucocorticoids are responsible for modifying the immune response [51]. With respect to the hypothesis that inflammation induces sleep changes, one review suggests that IL-6 is the key factor [120]; however, we need to consider that IL-6 has been proposed as a putative sleep factor and is produced by nonimmune cells [121]. We agree that there is enough evidence to conclude that inflammation modifies sleep and that sleep loss modifies circulating cytokines. If we work with the hypothesis that proinflammatory cytokines induce sleep, then we may have found a natural condition in which there is a very high level of inflammation (e.g., sepsis) and test whether sleep is changed. There are some reviews on sepsis and sleep that show that patients with sepsis present increased non-REM sleep and decreased REM sleep, with high levels of cytokines, such as TNF and IL-1*β*, and show an altered EEG with low-voltage, mixed-frequency waves with variable theta and delta (“septic encephalopathy”) and also loss of normal circadian melatonin secretion [122]. Then, we could conclude that pro-inflammatory cytokines induce non-REM sleep. However, septic encephalopathy is not sleep, it is a sleep disorder, and melatonin has been successfully used in septic patients (reviewed in [122]). Thus, we come back to our hypothesis: the function of sleep is to maintain the integrity of the neuro-immune-endocrine system [11]. In this review we observe how diseases or inflammation can disrupt that integrity, and the organism will respond by modulating sleep to restore the homeostasis and also how sleep loss induces a disruption of the integrity of neuro-immuno-endocrine system causing an inadequate immune response.

## Figures and Tables

**Table 1:** Differential effect of sleep loss time upon glucocorticoid levels.

Human	Cortisol	Reference
TSD 1 night	↑	[34]
TSD 40 hours	= or ↓	[40]
TSD 40 hours	=	[39]
SR 2 hours TIB/1 night	=	[36]
SR 3 hours TIB/4 days	↓	[37]
SR 6 hours TIB/6 days	=	[41]

Rodents	Costicosterone	Reference

RSD 72 hours	↑	[32]
RSD 96 hours	↑	[33]
RSR with 6 hours of SO/21 days	=	[33]

The table illustrates the differential effect of acute sleep deprivation and sleep restriction upon glucocorticoid levels. Representative samples present in this table were measured within the first four hours after wakefulness in humans or at the beginning of the light phase in rodents.

Abbreviations: TSD: total sleep deprivation; SR: sleep restriction; TIB: time in bed; RSD: REM sleep deprivation; SO: sleep opportunity; ↑: increase; =: not change; ↓: decrease*.*

**Table 2:** Sleep loss effects on immune cellular components in humans.

Sleep loss condition	Subject's characteristics	Cells		Reference country
Sleep deprivation	11 males	Leukocyte ↑*	B lymphocytes =	[73] Brazil
2 nights	19–29 years	Neutrophil ↑**	T lymphocytes =	
Sleep restriction	10 females	WBC ↑**	B lymphocytes =	[74] Belgium
4 hours time in bed	PM-RT	Monocytes ↑*	T lymphocytes =	
3 nights	55–65 years	Neutrophils ↑*		
Sleep restriction	7 females, 7 males	NK =	B lymphocytes =	[76] USA
4.5 hours time in bed	39–61 years	Monocytes =	T lymphocytes =	
1 night				
Sleep restriction	8 males	Neutrophils =	Lymphocytes =	[77] Belgium
4 hours time in bed	22–29 years			
3 nights				
Sleep restriction	13 males	Monocytes =	B lymphocytes ↑**	[75] Finland
4 hours time in bed	19–29 years	NK-cells↓**	T lymphocytes =	
5 nights				

The table illustrates the differences between sleep deprivation and sleep restriction upon cellular components of the immune system in humans.

Abbreviations: NK: natural killers; PM-RT: postmenopausal with replacement therapy; ↑: increase; =: not change; ↓: decrease; *significant differences with *P* < 0.05; **significant differences with *P* < 0.01.

**Table 3:** Sleep loss effects on immune molecular inflammatory mediators.

Sleep loss condition	Subject's characteristics	Cytokines (pg/mL)	C-reactive protein	Reference
Total sleep deprivation 1 night		IL-6 ↑ SL		[34] Germany
	16 controls	Control		
	11 females, 5 males	Basal 1.50 ± 1.10	ND	
	BMI 20.7–24.1 kg/m^2^	TSD 2.56 ± 1.63*	ND	
		Recovery 2.82 ± 1.94*	ND	
	15 unmedicated	Depressed		
	depressed patients	Basal 1.14 ± 0.69	ND	
	10 females, 5 males	TSD 2.38 ± 1.87*	ND	
	BMI 18.8–26.4 kg/m^2^			

Total sleep deprivation 1 night		IL-6 ? SL		[87] Italy
	9 females, 1 male	Basal ND	ND	
	Bipolar disorder	TSD 3.15 ± 5.14	ND	
	36–53 years			

Total sleep deprivation 40 hours		IL-1*β* ↑ PL	CRP ↑ PL mg/L	[40] USA
	9 females, 10 males	Basal ~0.20	Basal ~0.20	
	20–36 years	TSD ~0.45*	TSD ~0.50*	
	BMI 18.5–24.5 kg/m^2^	IL-6 ↑ PL		
		Basal ~1.6		
		TSD ~1.9*		

Total sleep deprivation 40 hours	12 healthy males 29.1 ± 3.3 years BMI 23.4 ± 1.5 kg/m^2^	IL-6 = Basal 0.60 ± 0.13 TSD 0.62 ± 0.10 Recovery 1.20 ± 0.23* TNF-*α* = Basal 0.88 ± 0.32TSD 1.05 ± 0.30	CRP = *µ*g/mL Basal 1.22 ± 0.46 TSD 0.55 ± 0.13 Recovery 0.61 ± 0.14	[88] France

Total sleep deprivation 40 hours		IL-6 = PL		[39] France
	12 healthy males 26–32 years	Basal ~3.5 TSD ~3.6	ND ND	
	BMI 21.9–24.9 kg/m^2^	TNF-*α* ↑ PL		
		Basal 0.66 ± 0.19		
		TSD 1.29 ± 0.33*		

Total sleep deprivation 88 hours			CRP ↑ PL mg/L	[89] USA
	10 healthy males		Basal 0.39 ± 0.13	
	22–37 years		Day 1: 0.48 ± 0.16*	
		ND	Day 2: 0.50 ± 0.20*	
			Day 3: 0.65 ± 0.23*	
			Recovery 0.66 ± 0.24*	

Sleep restriction 5 hours time in bed (1 night)		IL-6 ↑ PL		[90] Tunisia
	20 males	Basal 1.89 ± 0.06	ND	
	20–22 years 71–75 kg	SR 3.9 ± 0.70*	ND	

Sleep restriction 4.2 hours time in bed (2 nights)		IL-6 = PL		[84] Germany
	15 males	Basal 2.0 ± 0.0	ND	
	20–40 years BMI 20.5–24.9 kg/m^2^	SR 2.2 ± 0.02	ND	

Sleep		IL-6 ↑ PL		[91] USA
restriction	25 control males	Basal ~2.9	ND	
4 hours time in		SR ~2.8	ND	
bed (4 days)	25 alcoholic males	SR + Alc ~4.1	ND	
		TNF-*α* ↑ PL		
		Basal ~1.2		
		SR ~1.0		
		SR + Alc ~3.0		

Sleep restriction 1 hour time in bed (7 nights)		IL-1*β*↑ PL	CRP↑ PL mg/L	[92] Norway
	8 males	Basal 8.9 ± 2.8	Basal 1.38 ± 0.89	
	25.8 ± 0.9 years BMI 80 ± 3.7 kg/m^2^ Demanding physical challenges and SR	SR day 7: 45.2 ± 6.3*	SR Day 7: 11.38 ± 3.05*	

Sleep		ND	CRP ↑ PL mg/L	[89] USA
restriction	4 females, 6 males		Basal 0.51 ± 0.20	
4.2 hours time	26–38 years		SR 2.65 ± 1.31*	
in bed (10 nights)	BMI 21–31 kg/m^2^			

Sleep		IL-6↑ PL	CRP = SL mg/L	[85] USA
restriction	6 females, 12 males	Basal 1.88 ± 0.85	Basal 0.34 ± 0.27	
4 hours time in	21–40 years	SR D10: 3.04 ± 2.83*	SR Day 10: 0.69 ± 0.76	
bed (10 nights)	BMI 20–26 kg/m^2^	Recovery 2.36 ± 1.36*		

Sleep		IL-6 ↑ SL	CRP ↑ SL mg/L	[93] Iceland
fragmentation	22 females, 136 males			
OSA patients	BMI < 30 kg/m^2^	1.3 ± 0.1	1.8 ± 0.2	
	28 females, 136 males			
	BMI 30.1–34.9 kg/m^2^	1.6 ± 0.2**	4.1 ± 0.5**	
	25 females, 107 males			
	BMI > 35 kg/m^2^	2.2 ± 0.2**	2.6 ± 0.3**	

Sleep		TNF-*α* ↑ PL		[94] Spain
fragmentation	148 children	AHI ≤ 1: 3.30 ± 0.4		
OSA patients	6–12 years	AHI ≥ 10: 10.02 ± 1.36*	ND	

Sleep fragmentation veterans		IL-1*β* = PL		[95] USA
	Good sleep 7 males	Good sleep ~1.7	ND	
	Poor sleep 58 males	Poor sleep ~3.2	ND	
		IL-6 = PL		
		Good sleep ~37.6		
		Poor sleep ~34.2		
		
		TNF-*α* = PL		
		Good sleep ~0.8		
		Poor sleep ~1.2		

Sleep fragmentation OSA patients	Sleep durations <6 hours, 249 males 6-7 hours, 227 males >7 hours, 135 males	IL-6 = PL <6 hours: 2.08–2.54 6-7 hours: 1.96–2.39 >7 hours: 2.00–2.59 TNF-*α* = PL <6 hours: 2.86–3.73 6-7 hours: 2.52–3.28 >7 hours: 2.19–3.10	CRP ↑ SL mg/L <6 hours: 1.79–2.47 6-7 hours: 1.71–2.35 >7 hours: 1.71–2.56	[96] USA

Abbreviations: AHI: apnea-hypopnea index (expressed as the number of events per hour of total sleep time); BMI: body mass index; ND: nondetermined; OSA: obstructive sleep apnea; PL: plasma levels; SL: serum levels; SR: sleep restriction; TSD: total sleep deprivation; ~: approximate values obtained from report tables; ↑: increase; =: not change; ↓: decrease; ?: without basal data; *significant differences with *P* < 0.05; **significant differences with *P* < 0.01. Mean ± standard deviation.

## Abbreviations

APCs: Antigen-presenting cells
BMI: Body mass index
CRP: C-reactive protein
EEG: Electroencephalography
ET-1: Endothelin-1
HIV: Human immunodeficiency virus
HPA: Hypothalamus-pituitary-adrenal axis
hsCRP: High-sensitivity CRP
IFN: Interferon
ICAM: Intracellular adhesion molecule
ICV: Intracerebroventricular
IL: Interleukin
MIP: Macrophage inhibitory protein
NK: Natural killer
Non-REM: Nonrapid eye movement
OSA: Obstructive sleep apnea
REM: Rapid eye movement sleep
SWS: Slow-wave sleep
T_h_: T helper
TNF: Tumor necrosis factor
VGKC: Voltage-gated potassium channels
WBC: White blood cells.

## References

[1] Everson CA, Bergmann BM, Rechtschaffen A (1989). Sleep deprivation in the rat: III. Total sleep deprivation. *Sleep*.

[2] Siegel JM (2008). Do all animals sleep?. *Trends in Neurosciences*.

[3] Rector DM, Schei JL, Van Dongen HPA, Belenky G, Krueger JM (2009). Physiological markers of local sleep. *European Journal of Neuroscience*.

[4] Smith C (1995). Sleep states and memory processes. *Behavioural Brain Research*.

[5] Peigneux P, Laureys S, Delbeuck X, Maquet P (2001). Sleeping brain, learning brain. The role of sleep for memory systems. *NeuroReport*.

[6] Guzman-Marin R, Suntsova N, Bashir T, Nienhuis R, Szymusiak R, McGinty D (2008). Rapid eye movement sleep deprivation contributes to reduction of neurogenesis in the hippocampal dentate gyrus of the adult rat. *Sleep*.

[7] Meerlo P, Mistlberger RE, Jacobs BL, Craig Heller H, McGinty D (2009). New neurons in the adult brain: the role of sleep and consequences of sleep loss. *Sleep Medicine Reviews*.

[8] Gomez-Gonzalez B, Hurtado-Alvarado G, Esqueda-Leon E, Santana-Miranda R, Rojas-Zamorano JA, Velazquez-Moctezuma J (2013). REM sleep loss and recovery regulates blood-brain barrier function. *Current Neurovascular Research*.

[9] Schmid DA, Wichniak A, Uhr M (2006). Changes of sleep architecture, spectral composition of sleep EEG, the nocturnal secretion of cortisol, ACTH, GH, prolactin, melatonin, ghrelin, and leptin, and the DEX-CRH test in depressed patients during treatment with mirtazapine. *Neuropsychopharmacology*.

[10] Krueger JM, Obál F, Fang J (1999). Why we sleep: a theoretical view of sleep function. *Sleep Medicine Reviews*.

[11] Gomez-Gonzalez B, Dominguez-Salazar E, Hurtado-Alvarado G (2012). Role of sleep in the regulation of the immune system and the pituitary hormones. *Annals of the New York Academy of Sciences*.

[12] Banks S, Dinges DF (2007). Behavioral and physiological consequences of sleep restriction. *Journal of Clinical Sleep Medicine*.

[13] McCoy JG, Strecker RE (2011). The cognitive cost of sleep lost. *Neurobiology of Learning and Memory*.

[14] Velazquez-Moctezuma J, Salazar ED, Retana-Marquez S (1996). Effects of short- and long-term REM sleep deprivation on sexual behavior in male rats. *Physiology and Behavior*.

[15] Faraut B, Boudjeltia KZ, Vanhamme L, Kerkhofs M (2012). Immune, inflammatory and cardiovascular consequences of sleep restriction and recovery. *Sleep Medicine Reviews*.

[16] Reynolds AC, Banks S (2010). Total sleep deprivation, chronic sleep restriction and sleep disruption. *Progress in Brain Research*.

[17] Maggio M, Colizzi E, Fisichella A (2013). Stress hormones, sleep deprivation and cognition in older adults. *Maturitas*.

[18] McEwen BS (2006). Sleep deprivation as a neurobiologic and physiologic stressor: allostasis and allostatic load. *Metabolism*.

[19] Anisman H, Merali Z (1999). Understanding stress: characteristics and caveats. *Alcohol Research and Health*.

[20] Selye H (1936). A syndrome produced by diverse nocuous agents. *Nature*.

[21] Chrousos GP (1998). Stressors, stress, and neuroendocrine integration of the adaptive response the 1997 hans selye memorial lecture. *Annals of the New York Academy of Sciences*.

[22] Jensen MM (1969). Changes in leukocyte counts associated with vaous stressors. *RES Journal of the Reticuloendothelial Society*.

[23] Dhabhar FS, Miller AH, McEwen BS, Spencer RL (1995). Effects of stress on immune cell distribution: dynamics and hormonal mechanisms. *The Journal of Immunology*.

[24] Herbert TB, Cohen S (1993). Stress and immunity in humans: a meta-analytic review. *Psychosomatic Medicine*.

[25] Velazquez-Moctezuma J, Dominguez-Salazar E, Cortes-Barberena E (2004). Differential effects of rapid eye movement sleep deprivation and immobilization stress on blood lymphocyte subsets in rats. *NeuroImmunoModulation*.

[26] Dhabhar FS (2013). Psychological stress and immunoprotection versus immunopathology in the skin. *Clinics in Dermatology*.

[27] Dhabhar FS, McEwen BS (1997). Acute stress enhances while chronic stress suppresses cell-mediated immunity in vivo: a potential role for leukocyte trafficking. *Brain, Behavior, and Immunity*.

[28] Himmerich H, Fischer J, Bauer K, Kirkby KC, Sack U, Krugel U (2013). *Stress-Induced Cytokine Changes in Rats*.

[29] Saul AN, Oberyszyn TM, Daugherty C (2005). Chronic stress and susceptibility to skin cancer. *Journal of the National Cancer Institute*.

[30] Elenkov IJ (2004). Glucocorticoids and the Th1/Th2 balance. *Annals of the New York Academy of Sciences*.

[31] Kushida CA, Bergmann BM, Rechtschaffen A (1989). Sleep deprivation in the rat: IV. Paradoxical sleep deprivation. *Sleep*.

[32] Yehuda S, Sredni B, Carasso RL, Kenigsbuch-Sredni D (2009). REM sleep deprivation in rats results in inflammation and interleukin-17 elevation. *Journal of Interferon and Cytokine Research*.

[33] Zager A, Andersen ML, Ruiz FS, Antunes IB, Tufik S (2007). Effects of acute and chronic sleep loss on immune modulation of rats. *American Journal of Physiology*.

[34] Voderholzer U, Fiebich BL, Dersch R (2012). Effects of sleep deprivation on nocturnal cytokine concentrations in depressed patients and healthy control subjects. *The Journal of Neuropsychiatry and Clinical Neurosciences*.

[35] Leproult R, Copinschi G, Buxton O, Van Cauter E (1997). Sleep loss results in an elevation of cortisol levels the next evening. *Sleep*.

[36] Faraut B, Boudjeltia KZ, Dyzma M (2011). Benefits of napping and an extended duration of recovery sleep on alertness and immune cells after acute sleep restriction. *Brain, Behavior, and Immunity*.

[37] Wu H, Zhao Z, Stone WS (2008). Effects of sleep restriction periods on serum cortisol levels in healthy men. *Brain Research Bulletin*.

[38] DeFranco DB (2002). Functional implications of glucocorticoid receptor trafficking. *Ernst Schering Research Foundation workshop*.

[39] Chennaoui M, Sauvet F, Drogou C (2011). Effect of one night of sleep loss on changes in tumor necrosis factor alpha (TNF-*α*) levels in healthy men. *Cytokine*.

[40] Frey DJ, Fleshner M, Wright KP (2007). The effects of 40 hours of total sleep deprivation on inflammatory markers in healthy young adults. *Brain, Behavior, and Immunity*.

[41] Pejovic S, Basta M, Vgontzas AN (2013). The effects of recovery sleep after one workweek of mild sleep restriction on Interleukin-6 and cortisol secretion and daytime sleepiness and performance. *American Journal of Physiology Endocrinology and Metabolism*.

[42] Vázquez-Palacios G, Retana-Márquez S, Bonilla-Jaime H, Velázquez-Moctezuma J (2001). Further definition of the effect of corticosterone on the sleep-wake pattern in the male rat. *Pharmacology Biochemistry and Behavior*.

[43] Born J, DeKloet ER, Wenz H, Kern W, Fehm HL (1991). Gluco- and antimineralocorticoid effects on human sleep: a role of central corticosteroid receptors. *American Journal of Physiology*.

[44] Bradbury MJ, Dement WC, Edgar DM (1998). Effects of adrenalectomy and subsequent corticosterone replacement on rat sleep state and EEG power spectra. *American Journal of Physiology*.

[45] Machado RB, Suchecki D, Tufik S (2005). Sleep homeostasis in rats assessed by a long-term intermittent paradoxical sleep deprivation protocol. *Behavioural Brain Research*.

[46] Mueller AD, Pollock MS, Lieblich SE, Epp JR, Galea LAM, Mistlberger RE (2008). Sleep deprivation can inhibit adult hippocampal neurogenesis independent of adrenal stress hormones. *American Journal of Physiology*.

[47] Tiba PA, De Menezes Oliveira MG, Rossi VC, Tufik S, Suchecki D (2008). Glucocorticoids are not responsible for paradoxical sleep deprivation-induced memory impairments. *Sleep*.

[48] Suchecki D, Tufik S (2000). Social stability attenuates the stress in the modified multiple platform method for paradoxical sleep deprivation in the rat. *Physiology and Behavior*.

[49] Imeri L, Opp MR (2009). How (and why) the immune system makes us sleep. *Nature Reviews Neuroscience*.

[50] De Simoni MG, Imeri L, De Matteo W, Perego C, Simard S, Terrazzino S (1995). Sleep regulation: interactions among cytokines and classical neurotransmitters. *Advances in Neuroimmunology*.

[51] Besedovsky L, Lange T, Born J (2012). Sleep and immune function. *Pflugers Archiv European Journal of Physiology*.

[52] Hori T, Katafuchi T, Take S, Shimizu N (1998). Neuroimmunomodulatory actions of hypothalamic interferon-*α*. *NeuroImmunoModulation*.

[53] Kovac A, Erickson MA, Banks WA (2011). Brain microvascular pericytes are immunoactive in culture: cytokine, chemokine, nitric oxide, and LRP-1 expression in response to lipopolysaccharide. *Journal of Neuroinflammation*.

[54] Dinarello CA (1996). Biologic basis for interleukin-1 in disease. *Blood*.

[55] Gemma C, Imeri L, De Simoni MG, Mancia M (1997). Interleukin-1 induces changes in sleep, brain temperature, and serotonergic metabolism. *American Journal of Physiology*.

[56] Dantzer R (2001). Cytokine-induced sickness behavior: where do we stand?. *Brain, Behavior, and Immunity*.

[57] Bacher N, Raker V, Hofmann C (2013). Interferon-*α* suppresses cAMP to disarm human regulatory T cells. *Cancer Research*.

[58] Bhopale MK, Hilliard B, Constantinescu CS (2013). DAB_389_IL-2 suppresses autoimmune inflammation in the CNS and inhibits T cell-mediated lysis of glial target cells. *Experimental and Molecular Pathology*.

[59] Lunde HM, Bjorvatn B, Myhr KM, Bo L (2013). Clinical assessment and management of sleep disorders in multiple sclerosis: a literature review. *Acta Neurologica Scandinavica*.

[60] Palesh O, Peppone L, Innominato PF (2012). Prevalence, putative mechanisms, and current management of sleep problems during chemotherapy for cancer. *Nature and Science of Sleep*.

[61] Haroon E, Raison CL, Miller AH (2012). Psychoneuroimmunology meets neuropsychopharmacology: translational implications of the impact of inflammation on behavior. *Neuropsychopharmacology*.

[62] Cornelius JR, Pittock SJ, McKeon A (2011). Sleep manifestations of voltage-gated potassium channel complex autoimmunity. *Archives of Neurology*.

[63] Iranzo A, Graus F, Clover L (2006). Rapid eye movement sleep behavior disorder and potassium channel antibody-associated limbic encephalitis. *Annals of Neurology*.

[64] Taibi DM (2013). Sleep disturbances in persons living with HIV. *Journal of the Association of Nurses in AIDS Care*.

[65] Choi SM, Kim BC, Kweon SS (2012). Restless legs syndrome in people affected by leprosy. *Leprosy Review*.

[66] Brower KJ, Aldrich MS, Hall JM (1998). Polysomnographic and subjective sleep predictors of alcoholic relapse. *Alcoholism*.

[67] Irwin MR, Olmstead R, Valladares EM, Breen EC, Ehlers CL (2009). Tumor necrosis factor antagonism normalizes rapid eye movement sleep in alcohol dependence. *Biological Psychiatry*.

[68] Krueger JM (2008). The role of cytokines in sleep regulation. *Current Pharmaceutical Design*.

[69] Opp MR, Obal F, Krueger JM (1991). Interleukin 1 alters rat sleep: temporal and dose-related effects. *American Journal of Physiology*.

[70] Fang J, Wang Y, Krueger JM (1997). Mice lacking the TNF 55 kDa receptor fail to sleep more after TNF*α* treatment. *Journal of Neuroscience*.

[71] Born J, Lange T, Hansen K, Mölle M, Fehm H-L (1997). Effects of sleep and circadian rhythm on human circulating immune cells. *The Journal of Immunology*.

[72] Dimitrov S, Lange T, Nohroudi K, Born J (2007). Number and function of circulating human antigen presenting cells regulated by sleep. *Sleep*.

[73] Ruiz FS, Andersen ML, Martins RCS, Zager A, Lopes JD, Tufik S (2012). Immune alterations after selective rapid eye movement or total sleep deprivation in healthy male volunteers. *Innate Immunity*.

[74] Kerkhofs M, Boudjeltia KZ, Stenuit P, Brohée D, Cauchie P, Vanhaeverbeek M (2007). Sleep restriction increases blood neutrophils, total cholesterol and low density lipoprotein cholesterol in postmenopausal women: a preliminary study. *Maturitas*.

[75] van Leeuwen WMA, Lehto M, Karisola P (2009). Sleep restriction increases the risk of developing cardiovascular diseases by augmenting proinflammatory responses through IL-17 and CRP. *PLoS ONE*.

[76] Irwin MR, Wang M, Ribeiro D (2008). Sleep loss activates cellular inflammatory signaling. *Biological Psychiatry*.

[77] Boudjeltia KZ, Faraut B, Stenuit P (2008). Sleep restriction increases white blood cells, mainly neutrophil count, in young healthy men: a pilot study. *Vascular Health and Risk Management*.

[78] Lange T, Perras B, Fehm HL, Born J (2003). Sleep enhances the human antibody response to hepatitis a vaccination. *Psychosomatic Medicine*.

[79] Benedict C, Brytting M, Markström A, Broman J-E, Schiöth HB (2012). Acute sleep deprivation has no lasting effects on the human antibody titer response following a novel influenza A H1N1 virus vaccination. *BMC Immunology*.

[80] Prather AA, Hall M, Fury JM (2012). Sleep and antibody response to hepatitis B vaccination. *Sleep*.

[81] Mills PJ, Von Känel R, Norman D, Natarajan L, Ziegler MG, Dimsdale JE (2007). Inflammation and sleep in healthy individuals. *Sleep*.

[82] Simpson NS, Banks S, Arroyo S, Dinges DF (2010). Effects of sleep restriction on adiponectin levels in healthy men and women. *Physiology and Behavior*.

[83] Dimitrov S, Lange T, Tieken S, Fehm HL, Born J (2004). Sleep associated regulation of T helper 1/T helper 2 cytokine balance in humans. *Brain, Behavior, and Immunity*.

[84] Schmid SM, Hallschmid M, Jauch-Chara K (2011). Disturbed glucoregulatory response to food intake after moderate sleep restriction. *Sleep*.

[85] Haack M, Kraus T, Schuld A, Dalal M, Koethe D, Pollmächer T (2002). Diurnal variations of interleukin-6 plasma levels are confounded by blood drawing procedures. *Psychoneuroendocrinology*.

[86] Klein-Wieringa IR, Andersen SN, Kwekkeboom JC (2013). Adipocytes modulate the phenotype of human macrophages through secreted lipids. *The Journal of Immunology*.

[87] Benedetti F, Lucca A, Brambilla F, Colombo C, Smeraldi E (2002). Interleukine-6 serum levels correlate with response to antidepressant sleep deprivation and sleep phase advance. *Progress in Neuro-Psychopharmacology and Biological Psychiatry*.

[88] Sauvet F, Leftheriotis G, Gomez-Merino D (2010). Effect of acute sleep deprivation on vascular function in healthy subjects. *Journal of Applied Physiology*.

[89] Meier-Ewert HK, Ridker PM, Rifai N (2004). Effect of sleep loss on C-Reactive protein, an inflammatory marker of cardiovascular risk. *Journal of the American College of Cardiology*.

[90] Abedelmalek S, Chtourou H, Aloui A, Aouichaoui C, Souissi N, Tabka Z (2013). Effect of time of day and partial sleep deprivation on plasma concentrations of IL-6 during a short-term maximal performance. *European Journal of Applied Physiology*.

[91] Irwin M, Rinetti G, Redwine L, Motivala S, Dang J, Ehlers C (2004). Nocturnal proinflammatory cytokine-associated sleep disturbances in abstinent African American alcoholics. *Brain, Behavior, and Immunity*.

[92] Gundersen Y, Opstad PK, Reistad T, Thrane I, Vaagenes P (2006). Seven days’ around the clock exhaustive physical exertion combined with energy depletion and sleep deprivation primes circulating leukocytes. *European Journal of Applied Physiology*.

[93] Arnardottir ES, Maislin G, Schwab RJ (2012). The interaction of obstructive sleep apnea and obesity on the inflammatory markers C-reactive protein and interleukin-6: the Icelandic Sleep Apnea Cohort. *Sleep*.

[94] El-Sheikh M, Buckhalt JA, Granger DA, Erath SA, Acebo C (2007). The association between children’s sleep disruption and salivary interleukin-6. *Journal of Sleep Research*.

[95] Guess J, Burch JB, Ogoussan K (2009). Circadian disruption, Per3, and human cytokine secretion. *Integrative Cancer Therapies*.

[96] Patel SR, Zhu X, Storfer-Isser A (2009). Sleep duration and biomarkers of inflammation. *Sleep*.

[97] Irwin MR, Wang M, Campomayor CO, Collado-Hidalgo A, Cole S (2006). Sleep deprivation and activation of morning levels of cellular and genomic markers of inflammation. *Archives of Internal Medicine*.

[98] Acosta-Rodriguez EV, Napolitani G, Lanzavecchia A, Sallusto F (2007). Interleukins 1*β* and 6 but not transforming growth factor-*β* are essential for the differentiation of interleukin 17-producing human T helper cells. *Nature Immunology*.

[99] Patel DN, King CA, Bailey SR (2007). Interleukin-17 stimulates C-reactive protein expression in hepatocytes and smooth muscle cells via p38 MAPK and ERK1/2-dependent NF-*κ*B and C/EBP*β* activation. *The Journal of Biological Chemistry*.

[100] Meier-Ewert HK, Ridker PM, Rifai N, Price N, Dinges DF, Mullington JM (2001). Absence of diurnal variation of C-reactive protein concentrations in healthy human subjects. *Clinical Chemistry*.

[101] Panagiotakos DB, Pitsavos C, Yannakoulia M, Chrysohoou C, Stefanadis C (2005). The implication of obesity and central fat on markers of chronic inflammation: the ATTICA study. *Atherosclerosis*.

[102] Okun ML, Luther JF, Wisniewski SR, Wisner KL (2013). Disturbed sleep and inflammatory cytokines in depressed and nondepressed pregnant women: an exploratory analysis of pregnancy outcomes. *Psychosomatic Medicine*.

[103] Pavon L, Sandoval-Lopez G, Eugenia Hernandez M (2006). Th2 cytokine response in major depressive disorder patients before treatment. *Journal of Neuroimmunology*.

[104] Shahsavand-Ananloo E, Berenji F, Sadeghniiat K (2013). Comparing effects of citalopram with fluoxetine on sleep quality in patients with major depressive disorder. *European Review for Medical and Pharmacological Sciences*.

[105] Arana-Lechuga Y, Nuñez-Ortiz R, Terán-Pérez G (2008). Sleep-EEG patterns of school children suffering from symptoms of depression compared to healthy controls. *World Journal of Biological Psychiatry*.

[106] Hernandez ME, Martinez-Fong D, Perez-Tapia M, Estrada-Garcia I, Estrada-Parra S, Pavón L (2010). Evaluation of the effect of selective serotonin-reuptake inhibitors on lymphocyte subsets in patients with a major depressive disorder. *European Neuropsychopharmacology*.

[107] Kudlow PA, Cha DS, Lam RW, McIntyre RS (2013). Sleep architecture variation: a mediator of metabolic disturbance in individuals with major depressive disorder. *Sleep Medicine*.

[108] Nguyen KD, Fentress SJ, Qiu Y, Yun K, Cox JS, Chawla A (2013). Circadian gene bmal1 regulates diurnal oscillations of Ly6C^hi^ inflammatory monocytes. *Science*.

[109] Druzd D, Scheiermann C (2013). Some monocytes got rhythm. *Science*.

[110] Arjona A, Sarkar DK (2005). Circadian oscillations of clock genes, cytolytic factors, and cytokines in rat NK cells. *The Journal of Immunology*.

[111] Keller M, Mazuch J, Abraham U (2009). A circadian clock in macrophages controls inflammatory immune responses. *Proceedings of the National Academy of Sciences of the United States of America*.

[112] Fortier EE, Rooney J, Dardente H, Hardy M-P, Labrecque N, Cermakian N (2011). Circadian variation of the response of T cells to antigen. *The Journal of Immunology*.

[113] Narasimamurthy R, Hatori M, Nayak SK, Liu F, Panda S, Verma IM (2013). Circadian clock protein cryptochrome regulates the expression of proinflammatory cytokines. *Proceedings of the National Academy of Sciences*.

[114] Weil ZM, Norman GJ, Karelina K (2009). Sleep deprivation attenuates inflammatory responses and ischemic cell death. *Experimental Neurology*.

[115] Vgontzas AN, Pejovic S, Zoumakis E (2007). Daytime napping after a night of sleep loss decreases sleepiness, improves performance, and causes beneficial changes in cortisol and interleukin-6 secretion. *American Journal of Physiology*.

[116] Stenholm S, Kronholm E, Bandinelli S, Guralnik JM, Ferrucci L (2011). Self-reported sleep duration and time in bed as predictors of physical function decline: results from the InCHIANTI study. *Sleep*.

[117] Breder CD, Dinarello CA, Saper CB (1988). Interleukin-1 immunoreactive innervation of the human hypothalamus. *Science*.

[118] Wisor JP, Schmidt MA, Clegern WC (2011). Evidence for neuroinflammatory and microglial changes in the cerebral response to sleep loss. *Sleep*.

[119] Zielinski R, Krueger JM (2011). Sleep and innate immunity. *Frontiers in Bioscience*.

[120] Rohleder N, Aringer M, Boentert M (2012). Role of interleukin-6 in stress, sleep, and fatigue. *Annals of the New York Academy of Sciences*.

[121] Vgontzas AN, Bixler EO, Lin H-M, Prolo P, Trakada G, Chrousos GP (2005). IL-6 and its circadian secretion in humans. *NeuroImmunomodulation*.

[122] Weinhouse GL, Schwab RJ (2006). Sleep in the critically ill patient. *Sleep*.

